# Discriminative Index: A Novel Indicator for Evaluating Machine Learning Algorithms in Laboratory Medicine

**DOI:** 10.3390/diagnostics16111671

**Published:** 2026-05-29

**Authors:** Hsin-Yao Wang, Wan-Ying Lin, Martin Spüler, Sven Brand, Jia-Ruei Yu, Markus Kostrzewa

**Affiliations:** 1 Microbiology and Infection Diagnostics, Bruker Scientific, Billerica, MA 01821, USA; hsin-yao.wang@bruker.com; 2 Department of Medicine, University at Buffalo-Catholic Health System, Buffalo, NY 14220, USA; wlin225@buffalo.edu; 3 Microbiology and Infection Diagnostics, Bruker Daltonics GmbH & Co. KG, 28359 Bremen, Germany; 4 Department of Laboratory Medicine, Chang Gung Memorial Hospital at Linkou, Taoyuan 333423, Taiwan

**Keywords:** machine learning, clinical decision support, gray zone, predictive probability distribution, distribution asymmetry, D-index, model evaluation, laboratory medicine

## Abstract

**Background**: Machine learning models are increasingly applied in clinical practice. However, conventional performance metrics such as sensitivity, specificity, and accuracy do not capture the structural characteristics of predictive probability distributions, limiting their utility for optimizing decision thresholds and supporting clinical decision-making. **Methods**: We propose the D-index, a novel metric designed to quantify asymmetry in predicted probability distributions by measuring the difference in skewness between positive and negative predictions. The D-index was evaluated using simulated datasets with skewness-dominant and kurtosis-dominant distributions at three performance levels (AUROC = 0.7, 0.8, and 0.9). Its practical utility was further validated using real-world MALDI-TOF mass spectrometry data for predicting vancomycin resistance in *Enterococcus faecium*, comparing three machine learning algorithms: deep learning, XGBoost, and random forest. **Results**: In simulated data, the D-index increased with model performance only in skewness-dominant distributions, demonstrating selective sensitivity to distributional asymmetry. In real-world data, the D-index distinguished models with similar conventional performance but different distributional structures. Models with higher D-index values retained a larger proportion of confidently classified cases while achieving meaningful accuracy improvements under a gray-zone strategy. In contrast, models with lower D-index values required exclusion of more cases to achieve comparable or greater accuracy gains. **Conclusions**: The D-index provides complementary information beyond traditional performance metrics by capturing structural properties of predictive distributions. It enables identification of models that are better suited for gray-zone strategies and supports more reliable clinical decision-making. This distribution-aware approach offers a practical tool for improving the safety, efficiency, and clinical utility of machine learning models in biomedical applications.

## 1. Introduction

Machine learning (ML) has achieved remarkable success across many fields, including laboratory medicine [1,2]. Although ML has been widely applied in laboratory medicine, several critical issues remain unresolved, including the lack of independent validation and suboptimal predictive performance. Traditionally, model performance has been evaluated using indicators such as sensitivity, specificity, and accuracy [3,4]. However, these conventional measures do not fully capture the practical utility of ML models, particularly in clinical medicine. In clinical applications, the accuracy of ML models typically ranges between 80% and 100%, which is far from perfect [5]. Given this limitation, the gray zone/indeterminate approach is one of the commonly employed methods to improve performance [6,7,8]. The gray zone is a well-established concept in laboratory medicine, where an indeterminate range is predefined. When a test result or prediction falls within this range, no definitive report is issued to clinicians. Only values outside the gray zone are reported. These values are considered more reliable. Reporting only reliable values helps reduce the risk of inappropriate clinical decision-making.

While the gray zone approach can significantly enhance diagnostic performance, it introduces a trade-off by reducing the proportion of cases with actionable results [6]. A wider gray zone improves reliability but simultaneously decreases the number of reported cases. At present, no standardized methodology exists for evaluating this trade-off. Furthermore, the boundaries of the gray zone are not necessarily symmetric around the cutoff; for example, they may be set as 0.45–0.60 rather than 0.50 ± 0.10 (i.e., 0.40–0.60). This flexibility makes the relationship between gray zone settings and case loss more complex, underscoring the need for a new evaluative tool.

To address this gap, we propose a novel performance indicator for ML models, the “discriminative-index” (D-index). The D-index is designed to complement traditional measures by identifying which algorithms are more suitable for enhancement through gray zone application. Specifically, the D-index is derived from the skewness of the distributions of positive and negative predictions: the greater the skewness of these distributions, the larger the D-index, indicating greater potential benefit from gray zone use.

In this study, we first verified the methodology of the D-index using simulated data. We then demonstrated its applicability with real-world matrix assisted laser desorption ionization-time of flight (MALDI-TOF) mass spectrometry data. Our results suggest that the D-index provides a valuable new metric for evaluating ML algorithms, particularly in contexts where gray zone strategies are needed to optimize clinical performance.

## 2. Methods

### 2.1. Study Design

**Figure 1:**
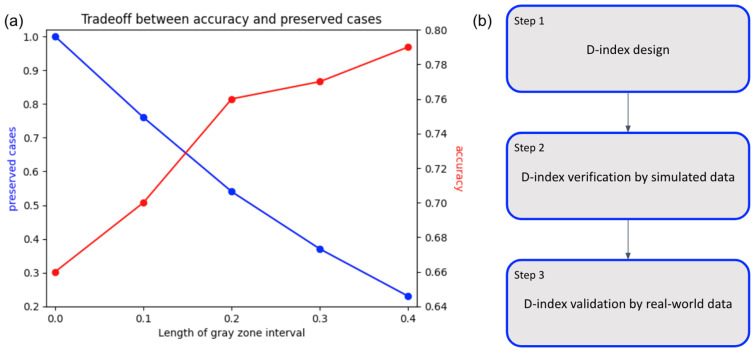
(**a**) Relation between gray zone and preserved cases. The figure illustrates the change in trends between preserved cases (blue color) and accuracy (red color). The preserved cases decrease when the size of the gray zone increases, indicating more invalid cases in the prediction. (**b**) Study design. To address the issue of picking up the most appropriate ML algorithm for the gray zone, the first step is to design D-index. In step 2 we verified the D-index by using different types of simulated data. In the last step, we validate the D-index with real-world data.

Figure 1 illustrates the process flowchart of this study. The left panel (Figure 1a) illustrates the idea of the trade-off between model performance (accuracy) and preserved cases in different levels of gray zones. The right panel (Figure 1b) shows the workflow of the study, including D-index design, D-index verification by simulated data, and D-index validation by real-world data.

**Figure 2:**
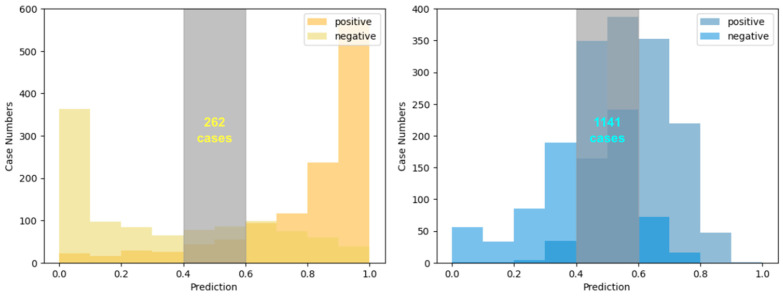
Illustration of applying a gray zone on two ML algorithms with similar original performance. Case numbers that are compensated from reporting are different between different ML algorithms even though their diagnostic performance is similar (in terms of accuracy). In the example, the predictions in the ML algorithm (left part) are distributed in a more skewed manner. By contrast, the predictions in the right part are distributed with a bell-like shape and concentrated around the cutoff (0.5). More cases are trimmed off from reporting when a gray zone is applied on the ML algorithms, whereas 262 cases are not trimmed off in the left side algorithm while 1141 cases in the right side algorithm.

In the step of D-index design, skewness and kurtosis were considered as the basic characteristics of the distributions for both positive and negative predictions (Supplement Figure S1). Theoretically for an ideal ML algorithm that is suitable for applying gray zone, the cases whose predictive probabilities close to the cutoff should be as less as possible so as to reduce the number of lost cases. Figure 2 illustrates an example of applying a gray zone to two ML algorithms with similar original performance. The two ML algorithms reveal very different distributions of predictive probabilities although their original performance is similar. However, significantly more cases (1141 cases) were trimmed off in the right-side algorithm (blue color) than the left-side algorithm (yellow color, 262 cases) under the same gray zone. The difference highlights the need for an additional index beyond traditional model metrics to determine which algorithm performs better in the gray zone. In the preliminary trials, we selected skewness as the fundamental characteristic for designing the D-index.

In step 2, we created three simulated datasets with different levels of model performance (i.e., area under the receiver operating characteristic curve (AUROC) = 0.9, 0.8, and 0.7). Using the datasets, we tested the performance of D-index in discriminating different ML algorithms. When the performance is compatible with our expectation, we tested D-index with a real-world biomedical dataset in step 3.

### 2.2. Design of the D-Index

The D-index quantifies the difference in distributional characteristics between the prediction scores of positive and negative classes in a ML model. Specifically, it measures the asymmetry and peakedness of these score distributions, reflecting how distinctly the model separates positive from negative predictions. Briefly, the D-index is generated by the following steps: (1) generation of predicted probabilities from the trained classification model, (2) separation of prediction scores according to the true class labels, (3) calculation of skewness for the positive and negative prediction distributions, and (4) computation of the D-index as the difference between the two skewness values.

Mathematically, the D-index is defined as the difference in skewness between the positive and negative prediction score distributions:

*D* = *S*_neg_ − *S*_pos_

where *S*_pos_ and *S*_neg_ represent the skewness of the predictive probability distributions for the positive and negative classes, respectively. The skewness was calculated with Fisher-Pearson coefficient of skewness, shown as below:

$$S=\frac{m_{3}}{m_{2}^{3/2}}$$

where

$$m_{i}=\frac{1}{N}\sum_{n=1}^{N}{\left(x[n]-\bar{x}\right)}^{i}$$

is the biased sample i-th central moment, and $\bar{x}$ is the sample mean. Skewness in this study was calculated using the scipy library (scipy.stats.skew) in Python (version 1.16.3).

The detailed characteristics of the D-index are described as below:

- Boundaries and Range: Because skewness has no upper or lower bound, there is no upper or lower bound to D-index. Specifically, a larger D-index value indicates greater asymmetry between class-specific prediction-score distributions.
- Distributional Behavior: The D-index is sensitive to the ‘tails’ of the predictive score distributions. In ML models, as the model becomes more ‘certain’ about its predictions, the scores tend to cluster toward 0 or 1, often resulting in increased skewness. The D-index quantifies the structural divergence of these clusters. Unlike the AUROC, which only considers the rank-order, the D-index reflects the probability density shift—specifically how the model’s confidence manifests differently across classes.
- Statistical Properties: As the D-index is derived from standardized third moments, it is invariant to linear transformations of the prediction scores, making it robust for comparing models with different output scales. In addition, the D-index specifically isolates the shape-related differences (asymmetry) between classes, providing an orthogonal dimension of model evaluation that complements traditional performance metrics.
- Interpretation: A larger D-index indicates greater disparity between the shapes of the two distributions. This disparity implies that the model’s predictions are more polarized and therefore more suitable for implementing a gray zone approach, where uncertain cases can be systematically excluded to enhance reliability. Conversely, a smaller D-index suggests that the positive and negative distributions overlap substantially near the cutoff and applying a gray zone would trim off too many cases.

In essence, the D-index serves as a diagnostic tool for understanding the structural characteristics of model outputs. By capturing higher-order distributional differences beyond mean and variance, it provides an additional layer of insight into model performance and its potential for clinical optimization through gray zone strategies.

### 2.3. D-Index Verification by Simulated Data

Simulated data was created to verify the idea of the d-index. Two types of probability distributions at three performance levels were adopted to create the simulated data. Specifically, the two types included the distributions with high skewness property (left panel of Figure 2) and the distributions with high kurtosis property (right panel of Figure 2). Skewness and kurtosis have been widely used in statistical learning to characterize the structure of predictive-score distributions [9]. This demonstrates that the distributional properties can carry meaningful separability information, which is relevant when determining whether a gray-zone strategy will improve model reliability. Three levels of predictive performance ranging from area under the receiver operating characteristic curve (AUROC) of 0.7 to 0.9 were adopted to cover the most common range of performance in the medical field [10,11].

**Figure 3:**
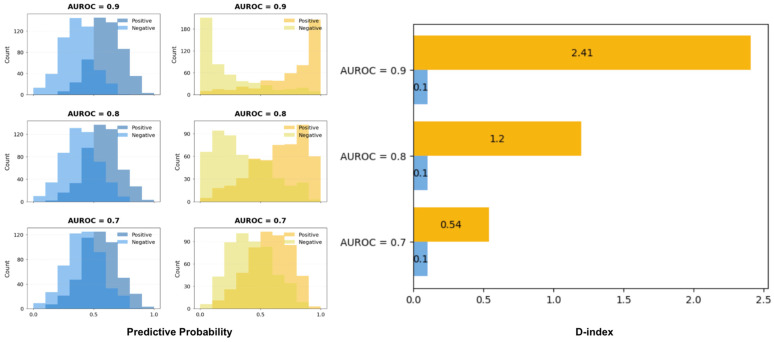
D-index scores in different levels of predictive performance. Three simulated datasets are used to demonstrate three levels of predictive performance (i.e., AUROC = 0.7, 0.8, and 0.9). For each level of predictive performance, the predictive distributions of the kurtosis-dominant algorithm (left side, blue color) and skewness-dominant algorithm (right side, yellow color) are plotted and their D-index scores are calculated (for the bar plot on the right panel, blue color stands for the kurtosis-dominant algorithms and yellow color for the skewness-dominant algorithms). The D-index scores of the skewness-dominant algorithm decrease gradually (from 2.41 to 0.54) when the predictions are less skewed. On the other hand, the D-index scores of the kurtosis-dominant algorithm stay at 0.1 for the predictive performance range from 0.7 to 0.9. The plots demonstrate that the D-index score is sensitive to the skewness level of the predictive distributions in a wide range of predictive performance (0.7–0.9). Moreover, the D-index score is able to discriminate the kurtosis-dominant algorithm from the skewness-dominant algorithm so as to determine the most appropriate algorithm to apply the gray zone.

Specifically for generating the simulated data, process was conducted as follows (source code can be found at the repository: https://github.com/imotwnsix/d-index (accessed on 31 March 2026)):

- Objective: For each target AUROC (0.7, 0.8, and 0.9), we generated two distinct models with divergent score distributions for the positive and negative classes.
- Model A (Blue distribution in Figure 3): The predictive scores were simulated using two Gaussian (normal) distributions for the positive and negative classes. The mean and variance were adjusted to achieve the precisely specified AUROC level.
- Model B (Yellow distribution in Figure 3): To create a contrasting distribution profile (representing a calibrated or ‘squashed’ probability range), we applied Platt scaling to the Gaussian-derived scores. This transformation altered the distribution shape and density, specifically shifting how scores are clustered around certain probability thresholds while mathematically maintaining the exact same AUROC as Model A.

On the basis of the simulated data sets, we calculated the D-index scores for the two predictive distributions at three performance levels.

### 2.4. D-Index Validation by a Real-World Dataset

We used real-world data from the medical field to test the utility of the proposed D-index after the verification. MALDI-TOF mass spectrometry data for predicting *Enterococcus faecium* resistance to vancomycin were used [12,13]. In total, the dataset included 5717 mass spectra (Linkou CGMH, 2795 resistant and 2922 susceptible), and another independent dataset included 2280 mass spectra of isolates (Kaohsiung CGMH, 1222 resistant and 1058 susceptible). The deep learning (DL), eXtreme Gradient Boosting (XGB), and random forest (RF) algorithms based models were developed based on the descriptions of the previous studies [12,13]. The raw predicted probability for each instance was collected and the distribution of the predicted probability was plotted as histograms. The D-index scores of the distributions of the models were calculated.

For the DL model, a one-dimensional Convolutional Neural Network (CNN) optimized for MALDI-TOF MS spectral analysis was implemented. The architecture began with an average pooling layer for initial signal processing, followed by three convolutional units. Each convolutional unit consisted of a convolutional layer, batch normalization, a rectified linear unit (ReLU) activation function, and an average pooling layer. The extracted features were subsequently flattened and connected to a 64-channel fully connected layer. Finally, a sigmoid output layer was used to generate predictive probabilities for binary classification. For the RF model, the hyperparameters included a maximum tree depth of 13, 350 decision trees (n_estimators = 350), and bootstrap sampling enabled (bootstrap = True). For the XGB model, the hyperparameters included gamma = 0.1, eta = 0.1, lambda = 2, maximum tree depth = 8, subsample ratio = 0.7, minimum child weight = 3, column sampling ratio per tree (colsample_bytree) = 0.7, and number of boosting rounds = 100. The model development procedures were based on our previously published framework [12,13] with additional analyses performed for the present study.

To ensure robust model evaluation and reduce the risk of overfitting, we implemented a validation framework combining internal cross-validation and external independent testing across all ML models, as described in the previous study [12,13]. For the primary dataset (Linkou CGMH), a 10-fold cross-validation strategy was applied for hyperparameter optimization and evaluation of model stability during development. After the optimal model configurations were identified, the final models were retrained using the complete Linkou CGMH dataset and subsequently validated using an independent hold-out testing dataset obtained from the Kaohsiung CGMH branch. Because the external testing cohort originated from a geographically distinct medical center, this validation strategy provided an additional safeguard against overfitting and enabled assessment of model generalizability across different clinical environments. Additional overfitting control measures were incorporated into the DL framework. Specifically, a dropout layer with a dropout rate of 0.5 was applied to the fully connected layers to reduce neuronal co-adaptation and improve generalization performance. In addition, the one-dimensional CNN architecture inherently reduced model complexity through parameter sharing within convolutional filters, thereby decreasing the total number of trainable parameters compared with conventional fully connected neural networks.

Raw predictive probabilities generated by each model were collected for subsequent D-index calculation and gray-zone analysis. Based on the D-index scores, we applied a gray zone (from 0.4 to 0.6) to the three models and compared the change in predictive accuracy and the trade-offs (cases that were missed in the gray zone). Cases with a predicted probability in the range of 0.4 to 0.6 were considered uncertain and therefore not reported as positive/negative, but as indeterminate.

## 3. Results

### 3.1. Performance of the D-Index in Simulated Datasets

The D-index was first evaluated using simulated data to examine its ability to discriminate between models with different distributional properties. Across all three performance levels (AUROC 0.7, 0.8, and 0.9), the simulated datasets successfully generated two distinct types of predictive probability distributions: one characterized by high skewness and the other by high kurtosis (left panel of Figure 3). Although these distributions were engineered to exhibit similar AUROC values, their structural shapes differed markedly, thereby providing a controlled environment to test the behavior of the D-index.

For each AUROC level, the D-index consistently produced higher values for the skewness-dominant distributions than for those dominated by kurtosis (Figure 3). This pattern aligned with our expectation that skewed distributions would yield clearer separation between positive and negative cases near the decision boundary. As a result, these distributions produced larger disparities in skewness between the two classes and therefore higher D-index values. In contrast, distributions with high kurtosis tended to show less pronounced class-specific asymmetry, resulting in lower D-index scores.

Importantly, the D-index values increased proportionally (from 0.54 for AUROC = 0.7 to 2.41 for AUROC = 0.9) with overall model performance in the skewness-dominant distributions only. In contrast, the D-index scores stayed as 0.1 for the kurtosis-dominant distributions across different AUROC levels.

### 3.2. Validation of the D-Index Using Real-World MALDI-TOF Data

**Figure 4:**
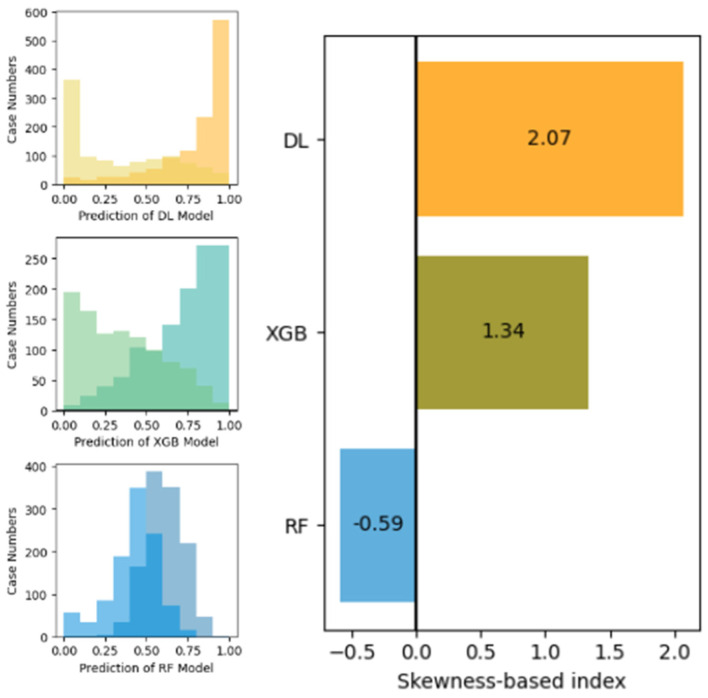
Distributional characteristics of prediction probabilities and corresponding D-index values for the evaluated models. (**Left** panel): Probability distributions of the deep learning (DL), extreme gradient boosting (XGB), and random forest (RF) models. For the plot of each model, the right-side distributions with darker color stand for the predictions of the positive cases and lighter color stand for the predictions of the negative cases. The DL and XGB models display skewness-dominant distributions, whereas the RF model shows a kurtosis-dominant pattern with greater overlap of class distributions around the decision cutoff. The DL model exhibits pronounced right-skewness among positive cases (darker yellow color) and left-skewness among negative cases (lighter yellow color), indicating stronger separation of the two classes. The XGB model shows moderate asymmetry between class distributions. In contrast, the RF model demonstrates substantial overlap between the two distributions near the cutoff. (**Right** panel): Corresponding D-index values for each model, quantifying the degree of asymmetry between class-specific prediction distributions. The DL model achieves the highest D-index (2.07), followed by the XGB model (1.34), while the RF model shows the lowest value (−0.59), indicating that DL would be the most appropriate algorithm to apply gray zone on.

The D-index was further evaluated using real-world MALDI-TOF mass spectrometry data for predicting vancomycin resistance in *Enterococcus faecium*. The probability distributions of the DL and XGB models showed skewness-dominant distributions while the distribution of the RF model revealed a kurtosis-dominant distribution (left panel of Figure 4). D-index scores were 2.07, 1.34, and −0.59 for DL, XGB, and RF models, respectively (right panel of Figure 4). The patterns and scores aligned with the results of the verification (Figure 3). The DL model produced a distribution with a pronounced right-skew among positive predictions and a strong left-skew among negative predictions, yielding the highest D-index among the algorithms. The XGB model exhibited moderately asymmetric distributions, resulting in an intermediate D-index. In contrast, the RF model showed substantial overlap between the two distributions near the cutoff, producing the lowest D-index.

**Figure 5:**
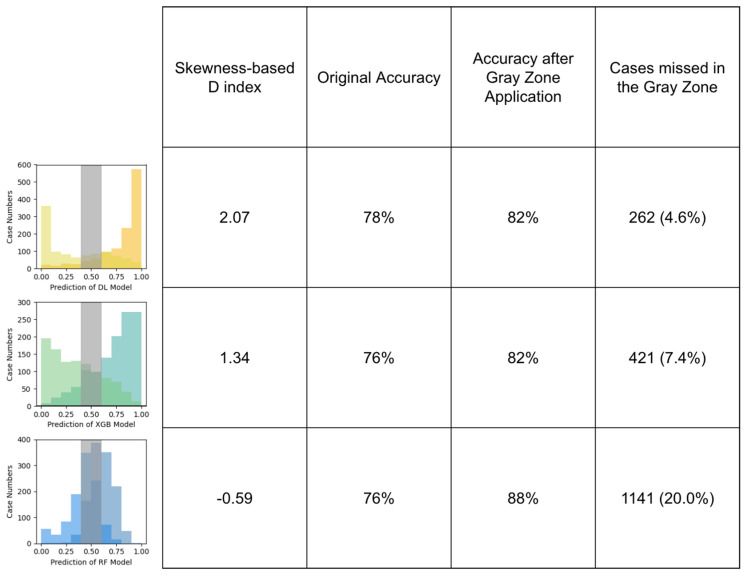
Impact of gray-zone application on model performance and case retention across models with different D-index values. Performance of the deep learning (DL), extreme gradient boosting (XGB), and random forest (RF) models before and after applying an identical gray-zone threshold. For the plot of each model, the right-side distributions with darker color stand for the predictions of the positive cases and lighter color stand for the predictions of the negative predictions. Prior to gray-zone exclusion, all three algorithms show comparable accuracy (DL: 78%, XGB: 76%, RF: 76%) despite differing D-index values (DL: 2.07, XGB: 1.34, RF: −0.59). After applying the gray zone, accuracy increases for all models (DL: 82%, XGB: 82%, RF: 88%). However, the number of excluded cases differs substantially. The RF model achieves the largest apparent improvement in accuracy but excludes the greatest number of cases within the gray zone (*n* = 1141). In contrast, the DL model, which had the highest D-index, retains the largest proportion of confidently classified cases while excluding the fewest (*n* = 262). The XGB model demonstrates intermediate behavior, with moderate improvement in accuracy and a moderate number of excluded cases (*n* = 421). These results illustrate how higher D-index values are associated with improved case retention when gray-zone strategies are applied.

To assess the practical utility of the D-index, a gray zone was applied to each model using identical thresholds (Figure 5). While all the three models had similar original accuracy (ranged from 76% to 78%), the D-index scores differed significantly (DL: 2.07, XGB: 1.34, RF: −0.59). After the gray zone was applied, the accuracy was elevated for all the models. DL model’s accuracy was elevated from 78% to 82%, XGB model’s accuracy was elevated from 76% to 82%, and RF model’s accuracy was elevated from 76% to 88%. While the RF model gained the largest improvement in accuracy, the RF model missed the highest number of cases in the gray zone (*n* = 1141). Consistent with the simulated findings, the DL model which had the highest D-index retained the largest proportion of confidently classified cases. The XGB model showed a moderate improvement in accuracy but excluded more cases than the DL model (421 vs. 262).

## 4. Discussion

In this study, we introduced and validated the D-index, a novel metric for quantifying the asymmetry of predictive probability distributions and demonstrated its relevance for selecting ML algorithms that are most amenable to gray-zone strategies. Our simulation experiments and real-world validation both show that the D-index is particularly sensitive to skewness in the distribution of predicted probabilities, and that models with higher D-index values behave more favorably when a gray zone is applied.

The proposed D-index discriminates skewness-dominant distribution from kurtosis-dominant distribution well. From our simulated data, we observed that as the overall discriminative performance (measured by AUROC) increased, the D-index rose markedly only in the skewness-dominant scenario, but remained nearly constant in the kurtosis-dominant scenario (Figure 3). This suggests that the D-index is not merely a proxy for overall discrimination but also responds specifically to the shape of the prediction distribution, particularly its asymmetry. In contrast, kurtosis-driven distributions did not produce increasing D-index values as performance improved (Figure 3). This distinction implies that models with symmetric or “sharp but symmetric” distributions may not benefit as much from gray-zone exclusion even if their discriminative performance is high.

The D-index reproduced the utility and worked well in a real-world setting. Our validation on real-world MALDI-TOF mass spectrometry data predicting vancomycin resistance in *Enterococcus faecium* (Figure 4) corroborated these simulation findings (Figure 3). With the highest D-index score, the DL model retained the largest proportion of confidently classifiable cases when a gray zone was applied (Figure 5). By contrast, the RF model showed a distribution with substantial overlap around the cutoff. RF model’s predictive probabilities were also less asymmetric than those of the other models. Because of these characteristics, the RF model produced the lowest D-index. When the gray-zone threshold was applied, it lost the largest number of cases (Figure 5). Notably, this occurred even though the RF model showed the greatest improvement in raw accuracy. These results empirically demonstrate that the D-index can predict which models will yield favorable trade-offs between improved classification accuracy and minimized loss of cases when implementing a gray zone. In the present study, the D-index does not define overall model superiority. Instead, it characterizes the trade-off between performance gain and case retention under gray-zone implementation. A model such as RF may be preferable in clinical settings where minimizing misclassification is the highest priority, even if more cases are excluded. By contrast, models with higher D-index values may be more desirable when preserving a larger proportion of evaluable cases is clinically important.

The broader significance of the D-index lies in its ability to fill an important gap in model evaluation that is often overlooked. Traditional metrics such as AUROC focus mainly on ranking performance, yet they do not describe the structure of the predictive probability distribution, nor offers guidance when a gray-zone is introduced. Recent work has also highlighted that calibration alone cannot fully solve this issue, because even a well calibrated model may generate score distributions that differ from the true risk distribution in real-world and may therefore be less useful in decision making [14]. Our findings emphasize that examining higher order characteristics of the predicted probability distribution, particularly skewness, can provide practical insight into how approaches such as gray zone exclusion may enhance the reliability and clinical usefulness of ML models.

To further evaluate the potential clinical relevance of the proposed metric, we additionally performed Decision Curve Analysis (DCA) by using pandas 2.2.2 and numpy 2.0.2. DCA is a widely used framework for assessing the net clinical benefit of predictive models across different decision thresholds [15]. In our analysis, the DCA results generally aligned with the overall predictive performance of the evaluated models (Supplement Figure S2). However, the D-index provided complementary information that was not directly reflected by DCA. Specifically, while DCA quantified the downstream clinical utility associated with different threshold selections, the D-index characterized the structural properties of predictive probability distributions and their suitability for gray-zone implementation. Models with higher D-index values tended to retain a larger proportion of confidently classified cases after gray-zone exclusion, despite having similar conventional performance metrics and comparable DCA profiles. These findings suggest that the D-index and DCA evaluate different but complementary aspects of model behavior. DCA focuses primarily on decision-level clinical benefit, whereas the D-index provides additional insight into how predictive uncertainty is distributed around the decision boundary. As a result, the D-index may serve as a useful supplementary tool when selecting models for workflows involving uncertainty exclusion or selective reporting.

Our findings align with growing recognition in the clinical prediction modeling community that model evaluation should extend beyond traditional discrimination metrics alone. Conventional measures such as AUROC primarily evaluate ranking performance and do not fully characterize the reliability or structural behavior of predictive probabilities. A tutorial published in the Journal of the American Medical Informatics Association emphasized that although AUROC remains a central evaluation metric, it “says nothing about the calibration of the model,” highlighting the importance of examining probability distributions and decision thresholds when models are intended for clinical use [16]. Recent discussions in the ML literature have similarly questioned overreliance on AUROC as a single summary measure, particularly in settings involving deployment shifts or clinically meaningful threshold selection [17,18]. In parallel, increasing attention has been directed toward calibration and uncertainty estimation in medical artificial intelligence. Studies on neural network calibration have shown that modern ML models may achieve excellent discrimination while still producing poorly calibrated or structurally misleading predictive probabilities [19,20]. Similarly, the MEMTAB symposium 2018 highlighted that traditional calibration measures may not fully capture distributional misalignment and recommended using more validation strategies [21]. Furthermore, emerging work on model based ROC analysis has demonstrated that the shape of predictive distributions and calibration characteristics can substantially influence the interpretation of ROC behavior itself [22]. The D-index provides a quantifiable measure of distributional asymmetry. This measure gives model developers and clinicians a clearer view of how the prediction scores are structured. With this information, they can better judge whether the use of a gray zone is appropriate. The D-index therefore serves as a practical tool that complements existing recommendations for model evaluation.

In the present study, the gray zone of 0.4 to 0.6 was selected as an illustrative example rather than as an optimized threshold for the specific prediction task. In clinical practice, the decision cutoff of binary classification models is commonly centered around 0.5. In addition, the greatest overlap between positive and negative predictive probability distributions is often concentrated near this decision boundary (Figure 5). For this reason, defining a symmetric gray zone around 0.5 provides an intuitive and clinically relevant demonstration of how uncertain predictions may be excluded in practice. We acknowledge, however, that gray-zone boundaries do not need to be symmetric and may vary according to clinical context, disease prevalence, and the relative consequences of false-positive and false-negative predictions. Different threshold settings would be expected to influence both the degree of performance improvement and the proportion of excluded cases. A systematic evaluation of threshold sensitivity, including asymmetric gray-zone definitions, would therefore be an important direction for future investigation.

A key limitation of the present study is that the real-world validation of the D-index was performed on one specific clinical task. Although the MALDI-TOF dataset used here is clinically relevant and sufficiently large for an initial evaluation, this design does not establish the generalizability of the proposed metric across broader laboratory medicine settings. Predictive probability distributions may vary substantially across institutions, patient populations, instruments, preprocessing pipelines, and disease contexts. As a result, the behavior and practical utility of the D-index may also differ under these conditions. Broader validation is therefore necessary before the D-index can be considered a general evaluation tool for clinical ML. Future studies should assess its performance across multiple centers, different organisms, diverse diagnostic applications, and other biomedical data modalities. Such validation will be essential to determine whether the observed relationship between distributional asymmetry and gray-zone performance is robust across different real-world settings.

There still are several limitations and considerations. First, the use of a gray zone also depends heavily on context. The width of the gray zone, the placement of its boundaries, and the acceptable trade-offs can vary across clinical settings. These choices depend on disease prevalence, risk tolerance, and the specific consequences of misclassification. Even models with a high D-index still require careful calibration and validation before clinical use. Inadequate threshold settings can lead to unsafe decisions. Threshold selection itself is an essential step in moving from predicted probabilities to actionable decisions. Prior work in Medical Decision Making has shown that default thresholds such as 0.5 are often inappropriate for clinical tasks. Alternative strategies, including Youden’s index and approaches based on clinical risk, should be considered. Another limitation is that our real-world validation focused on the datasets of a single biomedical topic. Although the datasets are both large and clinically meaningful, broader evaluation is needed to avoid overfitting. Future studies should test the D-index across a wider range of problems, including rare-event prediction, survival analysis, and settings with extreme class imbalance. Last, the D-index is designed to detect asymmetry in prediction distributions. The D-index is primarily designed to capture distributional asymmetry and may not adequately discriminate models whose main differences arise from kurtosis, multimodality, or other higher-order structural features. Our simulations supported this concern. In kurtosis-driven distributions, the D-index stayed nearly unchanged across performance levels. This pattern suggests that the D-index may fail to capture meaningful differences in those situations. The D-index should be interpreted alongside other established evaluation approaches rather than in isolation. Given these limitations, the proposal can be considered a supplementary contribution to studies on diagnostic validity.

## 5. Conclusions

In summary, our study presents the D-index as a novel, interpretable metric that captures predictive distribution asymmetry and helps identify models whose performance can be further optimized by gray-zone exclusion. By focusing on skewness rather than just predictive performance, the D-index fills a methodological gap in ML evaluation.

## Data Availability

The data presented in this study are available on request from the corresponding author due to institutional confidentiality and regulatory requirements.

## Supplementary Materials

The following supporting information can be downloaded at: https://www.mdpi.com/article/10.3390/diagnostics16111671/s1, Figure S1. Illustrative figures for the concepts of kurtosis and skewness. Figure S2. Decision curve analysis for VRE prediction models. Table S1. Comprehensive performance metrics.
