# Supplementary material for: Discriminative Index: A Novel Indicator for Evaluating Machine Learning Algorithms in Laboratory Medicine
Source: Diagnostics (Basel). 2026 May 29;16(11):1671. doi: 10.3390/diagnostics16111671 (PMC13256149; doi:10.3390/diagnostics16111671)
Supplement: Supplementary file 1 [file diagnostics-16-01671-s001.zip › diagnostics-4299237-supplementary.pdf]

Supplementary Materials

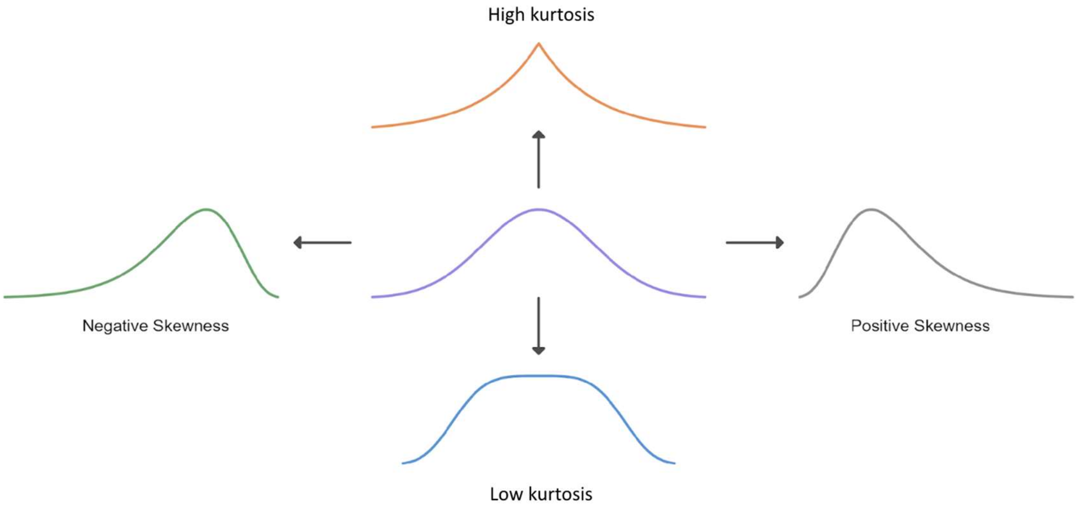

Supplement Figure S1. Illustrative figures for the concepts of kurtosis and skewness.

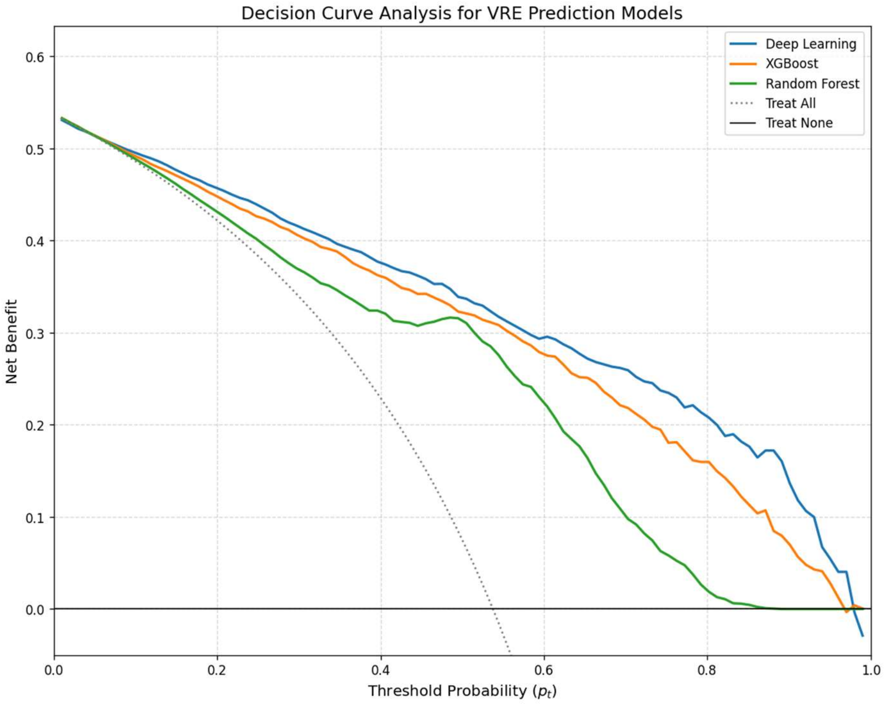

Supplement Figure S2. Decision curve analysis for VRE prediction models.

Supplement Table S1. Comprehensive performance metrics.

Model	D-Index	Original Accuracy	Original Precision	Original Recall	Original F1-Score	Accuracy After Gray Zone Application	Precision After Gray Zone Application	Recall After Gray Zone Application	F1-Score After Gray Zone Application	Cases Missed in the Gray Zone
DL	2.07	78%	75%	89%	81%	82%	79%	92%	85%	262
XGB	1.34	76%	76%	81%	79%	82%	81%	88%	84%	421
RF	0.59	76%	75%	83%	79%	88%	88%	94%	91%	1141
